# Plant Volatiles and Herbivore Induced Plant Volatiles from Chili Pepper Act as Attractant of the Aphid Parasitoid *Aphelinus varipes* (Hymenoptera: Aphelinidae)

**DOI:** 10.3390/plants11101350

**Published:** 2022-05-19

**Authors:** Muhammad Yasir Ali, Tayyaba Naseem, Jinping Zhang, Mingzhen Pan, Feng Zhang, Tong-Xian Liu

**Affiliations:** 1 Key Laboratory of Insect Ecology and Molecular Biology, College of Plant Health and Medicine, Qingdao Agricultural University, Qingdao 266109, China; y.ali@cabi.org (M.Y.A.); panmz2018@qau.edu.cn (M.P.); 2 MARA-CABI Joint Laboratory for Bio-Safety, Institute of Plant Protection, Chinese Academy of Agricultural Sciences, Beijing 100193, China; j.zhang@cabi.org; 3 Department of Botany, University of Agriculture Faisalabad, Faisalabad 38000, Pakistan; tayyaba_nasem@yahoo.com

**Keywords:** *Aphelinus varipes*, GCMS, *Myzus persicae*, plant volatiles, Y-tube olfactometer

## Abstract

Plants have evolved a number of different chemical defenses, covering nearly all classes of (secondary) metabolites, that represent a major barrier to herbivory: some are constitutive; others are induced after attacks from herbivores (HIPVs) and may elicit the attraction of predators and parasitoids. Here, we studied how the female solitary endoparasitoid *Aphelinus varipes* responds to plant and host aphid volatiles in a series of experiments on five commercially important vegetables that were either healthy or infested with the aphid *Myzus persicae*: chili pepper, eggplant, crown daisy, Chinese cabbage and cabbage. The results for the olfactory responses of *A. varipes* showed that the presence of *M. persicae* increased the attraction of the endoparasitoid to the infested plants. In a second experiment, volatiles from highly attractive and repellent plants were obtained via headspace collection to investigate volatiles from healthy and aphid-damaged plants. The results for the differences in volatile profiles in response to aphid infestation in chili pepper cultivar were dominated by the volatile blends, including α-pinene, decanal and phthalic acid, while in cabbage they were dominated by isophorone. Moreover, when HIPVs with different concentrations were compared, α-pinene at a dose rate of 100 ng/μL attracted more parasitoids, and the comparison was useful to understand the mechanisms of plant secondary volatiles during aphid infestation and to provide new resources to control this insect pest. Overall our study shows how HIPVs can bolster tritrophic interactions by enhancing the attractiveness of parasitoids.

## 1. Introduction

Plant volatiles play a profoundly important role in the structure and function of ecological communities [1]. Chemical defenses are a crucial mechanism through which plants can defend themselves against insect herbivores [2]. Plant defenses against insect herbivores are often divided into direct and indirect defenses [3]. Direct defenses act by themselves against the aggressor [4] and cause a negative impact on the development and behavior of the herbivore [5], whereas indirect defenses act via the attraction of organisms from an additional trophic level, e.g., natural enemies such as parasitoids, to exploit herbivores feeding on the plant [2,6,7,8]. In fact, the evolution and maintenance of the enormous diversity in plant secondary metabolites, particularly herbivore-induced plant volatiles (HIPVs), in relation to plant–insect interactions is one of the central topics in evolutionary ecology [9,10].

Undamaged plants emit traces of green leaf volatiles (GLVs), whereas comparatively higher amounts are rapidly released after damage, particularly upon herbivore feeding, which are thus known as herbivore-induced plant volatiles (HIPVs) and serve as long-range kairomones for natural enemies seeking hosts and prey [11]. However, HIPVs’ blend compositions are influenced by biotic factors such as the herbivore-infesting species, its infestation density and infesting instar and the host plant species, which in turn influence parasitoid responses [12,13]. HIPVs are perceived by plants, herbivores and natural enemies and consequently influence behavior on all three trophic levels [14].

The green peach aphid *Myzus persicae* Sulzer (Aphididae: Hemiptera) is a serious plant pest around the world due to its enormous host range (Asteraceae, Brassicaceae and Solanaceae families), immense reproductive capability, insecticide resistance [15] and ability to induce direct (sucking) and indirect (spreading of viruses and honeydew secretion) damage to plants [16,17]. The control of *M. persicae* exclusively relies on the use of synthetic insecticides such as organophosphates, neonicotinoids and pyrethroids [18]. These chemicals are simple and cost-effective; however, their tremendous use has triggered problems such as resistant behavior and environmental pollution, with negative side effects on human health [19]. Eco-friendly strategies to prevent such insect attacks on the final packaged product are therefore urgently required [20]. In response to insecticide resistance and environmental concerns and to promote ecofriendly strategies, the biological control of *M. persicae* and other aphid species using parasitoids, predators and entomopathogens has been adopted worldwide, both in greenhouses and in open fields [21,22].

The aphid solitary endoparasitoid *Aphelinus varipes* Foerster (Hymenoptera: Aphelinidae) is being used as a control measure against the Russian wheat aphid *Diuraphis noxia* (Mordwilko) in Australia [23] and the USA [24]; *M. persicae* in Canada [25] and China [26]; and *Rhopalosiphum padi*, *R. maidis* and *Macrosiphum euphorbiae* in other regions [27,28]. Parasitoids use HIPVs to locate their prey [29,30]; as indicated by Mohammed et al. (2020) [31], *Aphytis melinus* (DeBach; Hymenoptera: Aphelinidae) is significantly attracted towards HIPVs emitted by *Aonidiella aurantii*-fed citrus plants. The aim of the present study was thus to assess whether HIPVs can mediate the parasitoid behavior in the tritrophic interactions among *A. varipes*, *M. persicae* and host plants. We therefore hypothesized that HIPVs’ exposure may affect the attractiveness and olfaction of the *A. varipes* parasitoid as a prey of its host *M. persicae*.

To test this hypothesis, we studied the impact of constitutive and herbivore-induced volatiles on *A. varipes* using five commercial vegetable plants, with *M. persicae* and *A. varipes* were investigated differently with these five plants [26]: chili pepper (*Capsicum annum* L.; Solanaceae), eggplant (*Solanum melongena* L.; Solanaceae), crown daisy (*Chrysanthemum coronarium* Cass. ex Spach; Asteraceae), Chinese cabbage (*Brassica rapa* L.; Brassicaceae) and cabbage (*Brassica oleracea* L.; Brassicaceae). Our objective was to investigate whether plant volatile cues from healthy plants or those infested by *M. persicae* influence the responses of *A. varipes*. Initially, we observed the behavior of the female parasitoid in response to healthy plant volatiles and aphid-fed plant volatiles (HIPVs) by using a Y-tube olfactometer bioassay, which provided us with the more attractive (chili pepper) and repellent (cabbage) plant species. This was followed by chemical characterization of the volatile compounds released uniquely by infested plant seedlings compared with the uninfested seedlings through headspace collection, and those unique HIPVs were further tested in a Y-tube olfactometer. Overall, our experiments add a novel dimension to plant volatile-mediated tritrophic interactions, with implications for the ecology, evolution and potential applications of plant volatile-mediated tritrophic interactions.

## 2. Results

### 2.1. Bioassay of Olfactory Response of A. varipes towards Five Host Plants

**Figure 1:**
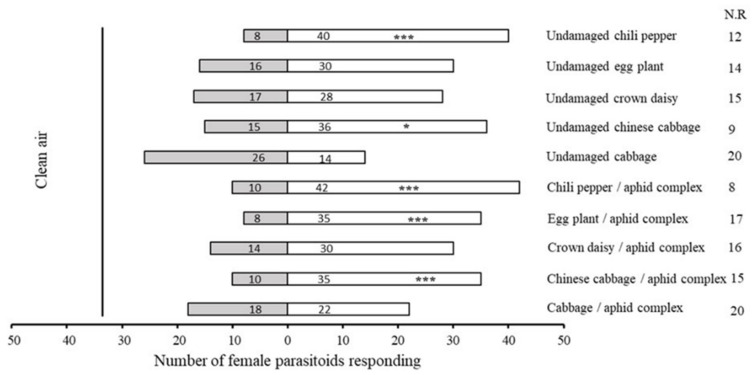
Olfactory responses of female *Aphelinus varipes* to the odors of five healthy plant species and the host–aphid complex compared to clean air. N.R indicates the non-responders’ female parasitoids. * *p* < 0.05, *** *p* < 0.0001, for χ^2^ test.

Chili pepper and cabbage exhibited strong and weak attraction towards parasitoids, respectively. Female adults of *A. varipes* preferred undamaged chili pepper (χ^2^ = 21.333, df = 1, *p* < 0.0001), undamaged Chinese cabbage (χ^2^ = 8.647, df = 1, *p* = 0.003), the chili pepper–aphid complex (χ^2^ = 19.692, df = 1, *p* < 0.0001), the Chinese cabbage–aphid complex (χ^2^ = 13.889, df = 1, *p* < 0.0001) and the eggplant–aphid complex (χ^2^ = 16.953, df = 1, *p* < 0.0001) to clean air. However, undamaged cabbage (χ^2^ = 3.6, df = 1, *p* = 0.58) and the cabbage–aphid complex (χ^2^ = 0.510, df = 1, *p* = 0.527) were as attractive as clean air for *A. varipes* females (Figure 1).

**Figure 2:**
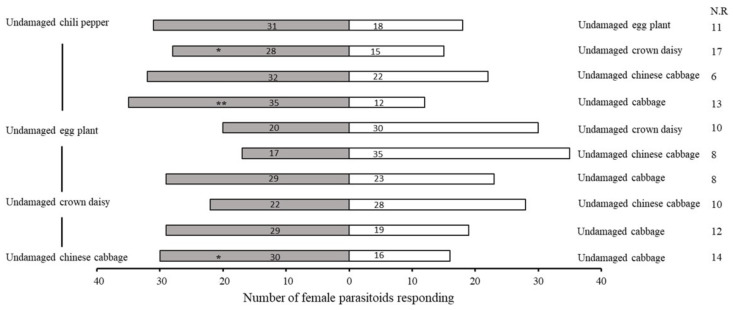
Olfactory responses of female *Aphelinus varipes* to the odors of undamaged host plants. N.R indicates the non-responders’ female parasitoids. * *p* < 0.05, ** *p* < 0.001, for χ^2^ test. An asterisk denotes a significant difference between odors tested.

**Figure 3:**
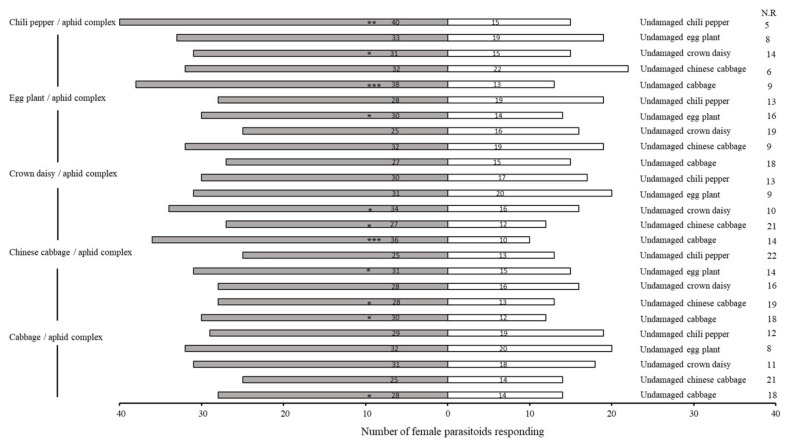
Olfactory responses of female *Aphelinus varipes* to the odors of different host/aphid complexes compared to undamaged host plants. N.R indicates the non-responders’ female parasitoids. * *p* < 0.05, ** *p* < 0.001, *** *p* < 0.0001, for χ^2^ test. An asterisk denotes a significant difference between odors tested.

Female parasitoids are usually attracted more by plant–aphid complexes than undamaged plants [32]. Volatiles from chili pepper–aphid plants attracted significantly more females as compared to healthy chili pepper seedlings (χ^2^ = 11.364, df = 1, *p* = 0.001). Similarly, the results for eggplant, crown daisy, Chinese cabbage and cabbage seedlings indicated that the plant–aphid complex attracted more parasitoids than undamaged samples (crown daisy–aphid complex: χ^2^ = 6.480, df = 1, *p* = 0.011; eggplant–aphid complex: χ^2^ = 5.818, df = 1, *p* = 0.016; Chinese cabbage–aphid complex: χ^2^ = 5.488, df = 1, *p* = 0.019; cabbage–aphid complex: χ^2^ = 4.667, df = 1, *p* = 0.031) (Figure 2 and Figure 3). Furthermore, *A. varipes* exhibited higher attraction for the chili pepper–aphid complex compared to undamaged cabbage (χ^2^ = 12.255, df = 1, *p* < 0.0001).

**Figure 4:**
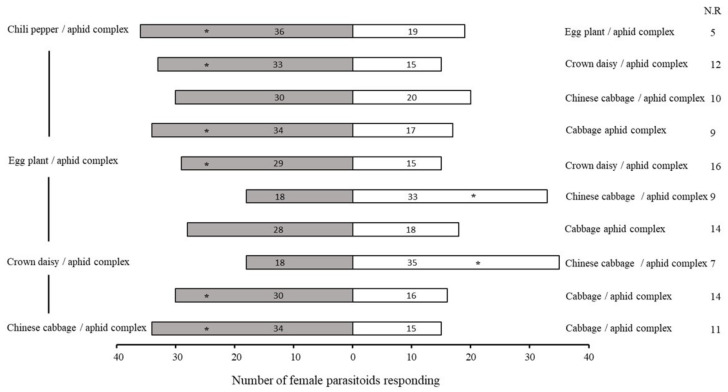
Olfactory responses of female *Aphelinus varipes* to the odors of aphid/plant complexes compared with aphid/plant complexes with other hosts. N.R indicates the non-responders’ female parasitoids. * *p* < 0.05, χ^2^ test. An asterisk denotes a significant difference between odors tested.

*Aphelinus varipes*, compared to the chili pepper–aphid complex and aphid complexes with other host plants, resulted in greater attraction (χ^2^ = 6.750, df = 1, *p* = 0.009), followed by the Chinese cabbage–aphid complex with the cabbage–aphid complex (χ^2^ = 7.367, df = 1, *p* = 0.007) (Figure 4). Moreover, both healthy (χ^2^ = 21.333, df = 1, *p* < 0.0001) and aphid-damaged chili pepper (χ^2^ = 6.750, df = 1, *p* < 0.009) attracted more parasitoids than other plants.

### 2.2. Identification of Volatiles Emitted from Healthy and Myzus Persicae-Induced Chili Pepper and Cabbage Plants

Gas chromatography mass spectrometry (GC-MS) analysis detected a total of 178 and 217 chemical compounds in the volatile samples collected from healthy chili pepper and aphid-fed chili pepper plants, respectively, and similarly 189 and 210 from healthy and aphid-fed cabbage plants, respectively. Quantitative analysis showed that volatiles including p-xylene, decane, benzene 1,4-diethyl, benzaldehyde 4-ethyl, p-cymen-7-ol, tetradecane 2,6,10-trimethyl, pentadecane and ethanone 1-(4-ethylphenyl) were released only from chili pepper and not the cabbage plant, which led to the attraction of parasitoid wasps among healthy seedlings (Table 1).

Furthermore, volatiles including 2,4-dimethyl-1-heptene, 3-methyl tridecane, 2,6,11-trimethyl dodecane, 1-hexadecanol and 1-nonadecene were only released from the healthy cabbage plant and not the healthy chili pepper, and they acted as repellents for the wasp (Table 2).

Three major compounds that were significantly present in plants infested with *M. persicae* were detected with GC-MS in chili pepper and one in cabbage. In contrast to all other compounds, emission of these herbivore-induced plant volatiles (HIPVs)—α-pinene, decanal and phthalic acid from chili pepper and isophorone from cabbage—was observed when plants were attacked by *M. persicae* (Table 3).

### 2.3. Bioassays with Synthetic Compounds

**Figure 5:**
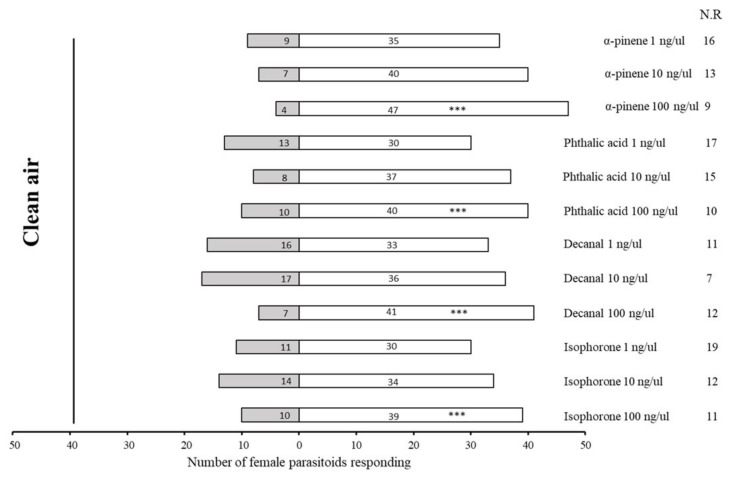
Olfactory responses of female *Aphelinus varipes* to the odors of four herbivore-induced plant volatiles (HIPVs). NR indicates non-responders’ female parasitoids. *** *p* < 0.0001, χ^2^ test.

The parasitoid *A. varipes* was more attracted to the highest of the HIPV doses tested for at 100 ng/μL (α-pinene: χ^2^ = 36.255, df = 1, *p* < 0.0001; decanal: χ^2^ = 24.083, df = 1, *p* < 0.0001; phthalic acid: χ^2^ = 18.00, df = 1, *p* < 0.0001; isophorone: χ^2^ = 17.163, df = 1, *p* < 0.0001) compared to clean air (Figure 5). Moreover, lower doses of one-tenth and one-hundredth of these attractant doses were also attractive to the parasitoid compared to clean air but not as much as 100 ng/μL.

**Figure 6:**
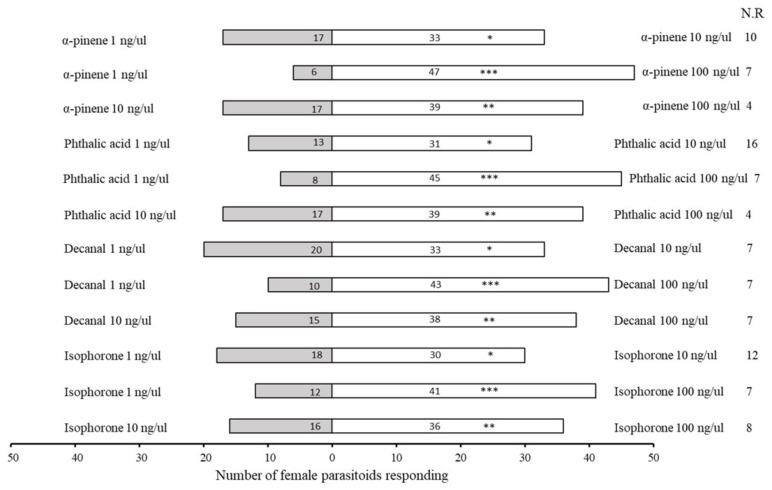
Test of preference of *Aphelinus varipes* toward four herbivore-induced plant volatiles (HIPVs) at three concentrations (1; 10; 100 ng/μL). An asterisk sign denotes a significant difference between odors tested (* *p* < 0.05, ** *p* < 0.001, *** *p* < 0.0001, χ^2^ test). N.R indicates non-responders’ female parasitoids.

The parasitoid *A. varipes* surprisingly preferred all HIPV chemicals at a higher dose rate (100 ng/μL); as the dose increased (1; 10; 100 ng/μL), the attractiveness also increased. The wasp showed the highest attraction for doses of 100 ng/μL, in the following order: α-pinene (χ^2^ = 31.717, df = 1, *p* < 0.0001), phthalic acid (χ^2^ = 25.830, df = 1, *p* < 0.0001), decanal (χ^2^ = 20.547, df = 1, *p* < 0.0001) and isophorone (χ^2^ = 15.868, df = 1, *p* < 0.0001) (Figure 6).

**Figure 7:**
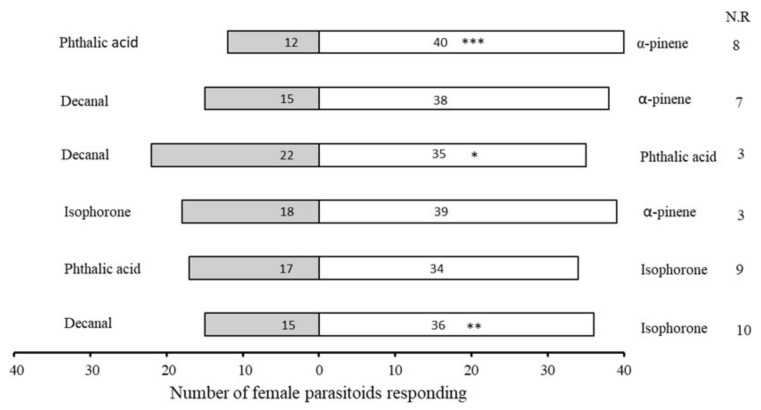
Olfactory responses of *Aphelinus varipes* to odors of four herbivore-induced plant volatiles (HIPVs) at 100 ng/ul concentrations. An asterisk denotes a significant difference between odors tested (* *p* < 0.05, ** *p* < 0.001, *** *p* < 0.0001, χ^2^ test). N.R indicates non-responders’ female parasitoids).

However, when given a choice between 100 ng/μL doses of HIPVs in a Y-tube olfactometer, *A. varipes* female parasitoids were significantly more attracted to α-pinene (χ^2^ = 15.077, df = 1, *p* < 0.0001) volatiles, followed by isophorone (χ^2^ = 8.647, df = 1, *p* = 0.003) and phthalic acid (χ^2^ = 2.965, df = 1, *p* = 0.085) (Figure 7).

## 3. Discussion

The results from this study indicate that parasitoids are attracted differently by different healthy plants, suggesting that some of them are more suitable for such a fit regardless of the presence of the aphid. The aphid-infested plants within the same species elicited more attraction from the parasitoids in comparison to healthy plants. The parasitoids were more attracted by certain plants infested by aphids rather than by other infested plants, suggesting that in these cases some HIPVs may be released (as the presence of the aphid is constant).

Herbivores’ natural enemies must locate their prey in a complex habitat consisting of multiple plant species damaged by different herbivores. Different species of natural enemies utilize different strategies to separate the signal from the noise contained in complex volatile organic compound (VOC) mixtures. In the present study, our results indicated that aphids’ natural enemies have the capability to distinguish the odors emitted by aphid–plant complexes and healthy seedlings. Naïve *Aphelinus varipes* showed a response to constitutive plant VOC chemicals and an even stronger response to aphid-induced VOCs, also called HIPVs. Furthermore, *A. varipes* females were strongly attracted by the odors of constitutive plant VOC chemicals emitted by chili pepper, irrespective of the constitutive plant VOC blends of the other plant species used in this study. Moreover, the parasitoid *A. varipes* showed more specific responses, preferring aphid-induced odors (HIPVs) but not plant volatiles. *Aphelinus varipes* wasps could differentiate between herbivore-induced plant volatiles from different plant species and were considerably attracted towards HIPVs from chili pepper rather than other HIPVs emitted from other aphid/plant complexes used in this study. These findings suggest the volatiles from plant/aphid complexes might facilitate the host foraging of *A. varipes*.

Volatiles from plant/aphid complexes can comprise chemicals from host aphids and aphid-induced compounds emitted by plants. The chemicals from aphids (e.g., chemicals present in the aphid cornicle, alarm pheromone, etc.) are essential for host acceptance and for the suitability of aphid parasitoids [50]. However, aphid-induced volatiles are highly detectable and reliable and play a key role in the process of locating a host among aphid parasitoids [51,52,53,54]. In this study, *A. varipes* responded at high levels towards the chili pepper–aphid complex and at a lower level towards the cabbage–aphid complex, suggesting the importance of volatiles among multiple plant/aphid complexes for attracting *A. varipes* [53].

We found that volatiles from healthy and *M. persicae*-infested plants were dominated by α-pinene, decanal, phthalic acid and isophorone, as previously reported in other studies, particularly [46,47,48,49]. However, in the host-foraging process, some primary plant volatiles may be utilized by parasitoids as attractants [55] and repellents [56]. For instance, some primary volatiles emanated by plants play a crucial role in attracting bio-control agents of phytophagous insects, including parasitic wasps [57]. Pan et al. [32] noted that the naïve parasitic wasp *Aphidius gifuensis* is significantly attracted towards volatile compounds (VOCs) emitted by pepper and repelled by cabbage VOCs.

Plants release a variety of different volatile compounds providing natural enemies with information that allows them to discriminate between host and non-host plants [58]. The results for the preliminary behavioral responses among fresh seedlings demonstrated that volatiles of chili pepper attracted significantly higher numbers of *A. varipes* than the other treatments and acted as attractants (Table 1) [59]. Furthermore, in our case the lower performance of the parasitoids towards cabbage was perhaps related to the presence of the worst plant chemical, which acted as a repellent for the parasitoids (Table 2) [60].

Differences in phylogenesis and ecology among aphids or host plants may induce different chemical blends [61]. However, *A. varipes* females were significantly attracted by aphid/plant complexes, irrespective of the original host plants. This suggests that *A. varipes* females may use similar strategies in employing volatiles for their host foraging and, moreover, it is possible that some universal chemicals attracting *A. varipes* may be present concurrently in plant/aphid complexes [61]. A universal chemical, α-pinene, prevailing in host aphid/chili pepper complexes can notably attract the important bio-control agent *A. varipes* against *M. persicae* green peach aphids [62].

Qualities and quantities of volatiles emitted from healthy plants or plant/herbivore complexes may differ or vary among host plant species [12,51,53,63]. Parasitoids can change their responses to different doses of volatiles based on their olfactory organs’ sensations during the foraging process [54,64]. The higher doses of HIPVs can result in significantly higher attraction among parasitoids [65,66], and in various cases the higher doses of HIPVs act as repellents as well [67]. Understanding the foraging behavior of parasitoids in response to chemical cues may be helpful in improving the effectiveness of their control in the field [52,68].

Since the initial discovery that HIPVs attract predators and parasitoids [69,70], a wealth of studies have investigated this phenomenon in detail and also in the context of possible solutions for pest control [71]. Although evidence that certain HIPVs can improve plant performance by attracting natural enemies is emerging [72], a number of studies also highlight the additional roles of HIPVs that may modulate their net fitness effects for plants, including within-plant priming [73], direct herbivore intoxication [74], herbivore repellence [75], herbivore attraction [6,76] and hyperparasitoid attraction [29]. However, to what extent HIPV-mediated tritrophic interactions actually benefit plants is a subject under debate. Our findings support the hypothesis that herbivore-induced plant volatiles (HIPVs) significantly attract natural enemies (e.g., parasitoids), which would ultimately benefit plants via tritrophic interactions with herbivores.

## 4. Conclusions

Our study provides direct evidence that herbivore-damaged plants are more attractive to *A. varipes* than healthy plants. The parasitoids preferred the volatiles of infested chili pepper plants over those of other infested plants, mainly due to the volatile differences in the plant HIPVs. Our findings show that the four aphid-induced volatiles (α-pinene, decanal, phthalic acid: chili pepper; isophorone: cabbage) elicit a strong attraction in parasitoids, and among these HIPVs, α-pinene at 100 ng/μL attracted significantly more wasps. These attractive compounds could thus be used to formulate a kairomone-based lure to enhance biological control and to complement other integrated pest management approaches for *M. persicae*. Specifically, the kairomone lure could be used to trap the parasitoids to be released in greenhouses and open fields for augmentative biological control of this pest in vegetable crops. Thus, herbivore-mediated effects of HIPVs should be considered in order to better understand the evolution and ecological complexity of tritrophic interactions in nature and to optimize the use of HIPVs in biological control.

## 5. Materials and Methods

### 5.1. Plants and Insects

**Figure 8:**
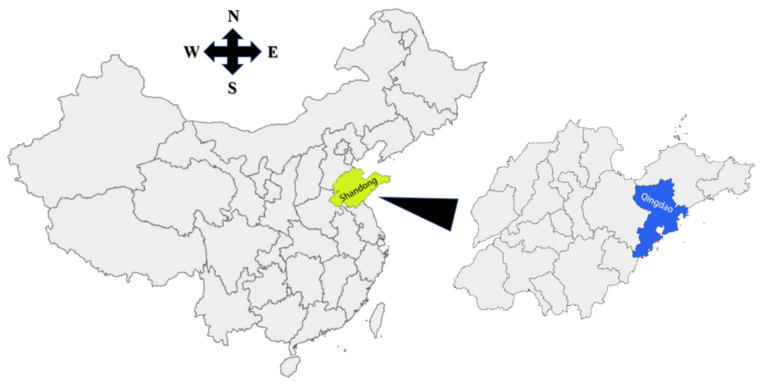
A map showing the study site (China, Shandong, Qingdao).

The green peach aphid *M. persicae* was taken from culture already reared in the lab as utilized by Ali et al. [5] and Ali et al. [26]. *Aphelinus varipes* parasitoids were collected by picking mummified immobile aphids from a field of Chinese cabbage in Qingdao (latitude: 36°03′57″ N; longitude: 120°22′09″ E; elevation above sea level: 46 m = 150 ft), in the eastern part of China, in 2018 (Figure 8). *Aphelinus varipes* was identified with a molecular method with COI as a marker (100% identical to sequence ID HQ599571.1) [77]. Colonies of aphids and parasitoids were maintained on plants grown in plastic pots (10 cm diameter) filled with soil mix (peat moss: perlite = 3: 1). Seedlings of about 15 cm height with 4–6 leaves were used for rearing and tests. *Myzus persicae* (insect pest) was bred on chili pepper (var. Japanese Chao tianjiao), eggplant (var. Guang jiazi hong chang qie), crown daisy (var. Xiao ye tong hao), Chinese cabbage (var. Zaoshu nan you xiao baicai) and cabbage (var. Jing feng yihao) plants separately in insect-rearing cages (40 × 40 × 30 cm^3^) (Figure S1). Plants were replaced in the aphid-rearing cages as needed due to the damage produced by aphids while feeding. *Aphelinus varipes* (parasitoid) was maintained in chili pepper plant–aphid associations and kept breeding until the whole experiment was finished. The blackish immobile mummified aphids were removed from these associations, separated into individual 1.5 mL tubes and saved in an incubator until emergence. Further, after emergence from these mummies, naïve one-day-old female specimens were tested in complete experiments. All plant and insect cultures were grown in an intelligent artificial climate chamber (Ningbo New Jiangnan Instrument Manufacturer Co., Ltd., Ningbo, China) at 25 ± 2 °C, with a 16: 8 h light: dark photoperiod and 65–75% RH, at the Key Laboratory of Insect Ecology and Molecular Biology of Qingdao Agriculture University, Shandong, China (Figure 8).

### 5.2. Y-Tube Olfactometer Bioassays

**Figure 9:**
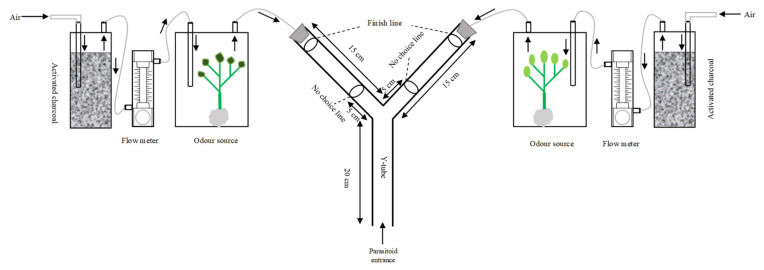
Schematic diagram of the Y-tube olfactometer used in this study.

All olfactory responses tests were carried out in a glass Y-tube olfactometer, following Pan et al. [32] with some modifications (Figure 9). The olfactometer (both the stem and the two arms were 15 cm in length, with an internal diameter of 1.4 cm) was used to investigate the response of the parasitoid *A. varipes* to the five plant species with and without aphid infestation. For each plant host, the olfactory response was determined using clean air, un-infested plants (healthy plants or fresh seedlings) and aphid-infested plants (plants infested with 100 mixed-instar aphids on seedlings for 72 h before the test).

Clean air was considered as a control unit and each plant species was tested before comparing them with each other. A curtain was settled around the onlooker to block light or other visual stimuli from entering from the exterior to the interior experimental setup. Experiments started around one hour after deposition of the plant sources in the glass chambers. In the experiments with plants, the roots and the base of the plant were tightly wrapped in aluminum foil to minimize interference with odors from the soil. The experiment was conducted in a dark room (25 ± 2 °C) with three 20 W fluorescent bulbs at 75 cm height above the Y-tube. Humidified air was pre-filtered through activated charcoal, drawn through two air flow meters and then pumped at 200 mL min^−1^ into two glass chambers that contained odor sources. Each adult naïve female parasitoid was introduced into the Y-tube from the entrance of the central stem. The parasitoid was given a limit of 5 min to make a choice, and the response was scored when it passed a line on the arm by approximately 5 cm and stayed there for not less than 30 s; otherwise, it was marked as a ‘no responder’ (time limit calculated with a Swiss CYMA Chronometer, c. 1910). Ten pairs of plants were tested in several combinations (Table S1). Each combination was tested with sixty parasitoids (a total of 60 × 55 = 3300), and the single female was considered a replicate and employed only once in the entire experiment. The orientation of the arms was reversed after testing 10 parasitoids. After testing 20 parasitoids, all glass chambers and Y-tubes were substituted with fresh ones. Only one treatment was tested per day to avoid the odor of one treatment affecting another test. The glass chambers and Y-tubes were cleaned with 75% ethanol and distilled water and then dried at 60 °C to minimize the effects of odor residues before they were used further. The experiments were carried out from 9 am to 4 pm.

### 5.3. Collection of Headspace Volatiles from Plants

**Figure 10:**
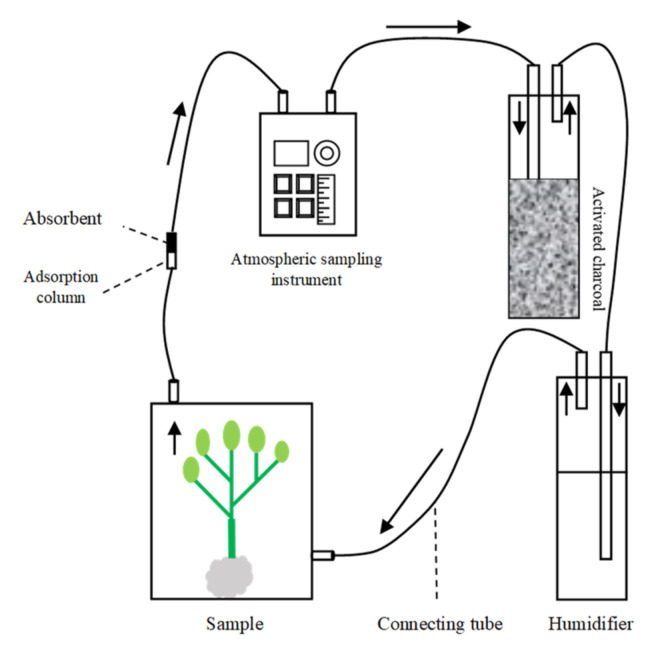
Schematic setup of dynamic headspace collection of plant volatiles.

Dynamic headspace collection was used to collect volatile compounds individually from fresh seedlings (uninfected plants) and plant–aphid complexes (infected with aphids) (Figure 10). Before the collection of volatiles, individual plants were prepared, and the roots and bases of the plants were tightly wrapped with aluminum foil in order to minimize the contamination of soil-based volatiles. To collect the volatiles, plants were enclosed in a glass collection chamber (2.5 L) (Analytical Research Systems, Inc., Gainesville, FL, USA) for two hours before the experiment was started. Air was pulled through the trapping filters using an air-sampling pump (Dymax 5, Charles Austen Pumps Ltd., West Byfleet, UK). The air was cleaned with an activated charcoal filter, pumped at a rate of 300 mL^−1^ and drawn by the glass chamber through a Teflon tube. Air containing headspace volatiles was pulled out of the glass chamber through a trapping filter (outlet fitted) containing 200 mg of Porapak-Q adsorbent (Waters Corporation, Milford, MA, USA). Each experimental unit consisted of three replications. After each collection, Porapak-Q filters containing the volatiles were eluted with 200 μL of HPLC-grade hexane (Sigma Aldrich, Beijing, China), which ultimately provided a solution containing volatile compounds. Further, the solution was concentrated to 20 μL under a nitrogen stream and stored at −80 °C in a sealed vial for future analysis. Before each collection of headspace volatiles, all the mandatory glass wares were rinsed with distilled water and acetone, and the filters were washed with 3 mL hexane. Volatile collection lasted for 6 h, and each collection was performed in the morning from 9 a.m. until 3 p.m. at room temperature (25 ± 2 °C) and under natural light conditions.

### 5.4. Analysis of Headspace Plant Volatiles in GC-MS

**Figure 11:**
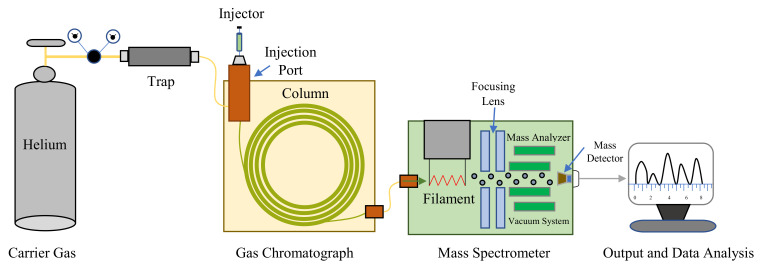
Schematic plot of the main components of the GC-MS instrument.

Plant volatiles from each treatment were collected for analysis and used in bioassays; i.e., the plants infected with aphids (aphid–plant complex) and the uninfected plants. Moreover, to identify the volatiles that were specifically released by the infested chili pepper and cabbage plants and that could promote attraction among herbivore natural enemies, the volatiles emitted by healthy plants and plants infested by *M. persicae* were compared. Volatiles extracted were analyzed with a Hewlett-Packard 7890A gas chromatograph coupled to a Hewlett-Packard 5975C mass selective detector (GC-MS) (Agilent Technologies, Santa Clara, CA, USA) with a HP-5MS capillary column (30 m × 0.25 mm × 0.25 μm) (Figure 11). The helium carrier gas used was at a flow rate of 1.3 mL^−1^. A 1 μL sample from 20 μL of standardized solution containing volatiles was made in split-less mode at an injector temperature of 230 °C and injected into the GC-MS. After injection, the temperature of the column was maintained at 75 °C for 2 min and afterward increased to 280 °C at 5 °C/min with an ending with 5 min holding time. An electronic ionization of 70 eV was used to obtain the mass spectra.

Volatile compounds were identified using GC-MS solution software (Shimadzu, ver. 2.53) by comparing their retention times and spectra with authentic standards [78]. For further identification of these compounds, the chemical identities of the existing peaks in the pod table aerations were tentatively assessed by comparing their mass spectra with those of the National Institute of Standards and Technology (NIST; Scientific Instrument Services, Gaithersburg, MD, USA) library and published mass spectra.

### 5.5. Chemicals

The chemical *α*-pinene (95% pure) was purchased from Tokyo Chemical Industry (Tokyo, Japan) and phthalic acid (99.5% pure) was from Shanghai Macklin Biochemical Co., Ltd. (Shanghai, China). Isophorone (97% pure) and decanal (98% pure) were from Shanghai Yuanye Biotechnology Co., Ltd. (Shanghai, China).

### 5.6. Bioassays with Synthetic Compounds

The attractiveness of the synthetic compounds α-pinene, decanal, phthalic acid and isophorone to parasitoid *A. varipes* (emerged from mummified aphids within 24 h) was tested using the above-described Y-tube olfactometer setup. Each compound was tested at three doses (1 ng/μL, 10 ng/μL and 100 ng/μL) and later on the compounds were compared with each other at a dose rate of 100 ng/μL. Hexane (solvent) was used to dilute the compounds, then an aliquot (10 μL) of the above-mentioned test solution was loaded onto 2 × 2 cm filter paper and tested (Figure S2). After 30 s to allow for the evaporation of the solvent, the impregnated filter papers were placed at the edge of the olfactometer arms and renewed for each insect. Sixty insects were tested per treatment (a total of 60 × 30 = 1800) (Table S2).

### 5.7. Statistics

IBM SPSS v. 26.0 (SPSS Inc., Chicago, IL, USA) was used to analyze the results obtained in this experiment. A chi-square test was used to separate the preferences of the female *A. varipes* among every paired combination of the five host plant species, and similarly for the bioassay with different chemical blends. Parasitoids that did not show a preference were excluded from the analysis before chi-square testing. All figures and graphs were produced in MS Office Package 2019 and illustrations were in Adobe Photoshop Package 2021.

## Figures and Tables

**Table 1:** Compositions of volatile blends collected in 6 h from undamaged chili pepper that acted as attractants.

No.	Retention Time (min)	Compound	Molecular Formula	Chemical Formula	Relative Level ± SE (%) *	Reference
1	3.89	p-xylene	C_8_H_10_	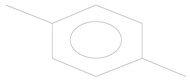	0.08 ± 0.006	[33]
2	6.59	Decane	C_10_H_22_	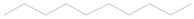	0.12 ± 0.012	[34]
3	7.99	Benzene, 1,4-diethyl	C_10_H_14_	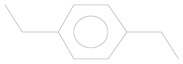	30.78 ± 0.577	[35]
4	11.10	Benzaldehyde, 4-ethyl-	C_9_H_10_O	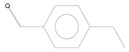	0.64 ± 0.012	[36]
5	13.77	p-cymen-7-ol	C_10_H_14_O	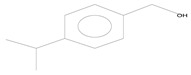	2.57 ± 0.028	[37]
6	13.97	Tetradecane, 2,6,10-trimethyl-	C_17_H_36_	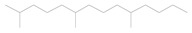	0.01 ± 0.009	[38]
7	19.71	Pentadecane	C_15_H_32_	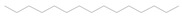	0.05 ± 0.006	[39]
8	25.51	Ethanone, 1-(4-ethylphenyl)-	C_10_H_12_O	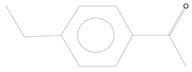	0.01 ± 0.006	[40]

* The percentage of relative level was calculated from 178 compounds detected with GC-MS and standard error (SE) was from three biological replications.

**Table 2:** Compositions of volatile blends collected in 6 h from undamaged cabbage plants that acted as repellents.

No.	Retention Time (min)	Compound	Molecular Formula	Chemical Formula	Relative Level ± SE (%) *	Reference
1	3.39	2,4-dimethyl-1-heptene	C_9_H_18_	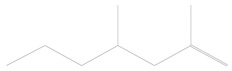	0.01 ± 0.010	[41]
2	16.50	3-methyl tridecane	C_14_H_30_	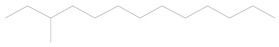	0.01 ± 0.006	[42]
3	16.62	2,6,11-trimethyl dodecane	C_15_H_32_	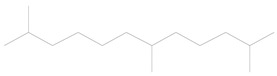	0.02 ± 0.006	[43]
4	21.90	1-hexadecanol	C_16_H_34_O	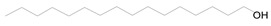	0.02 ± 0.006	[44]
5	24.14	1-nonadecene	C_19_H_38_	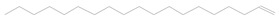	0.01 ± 0.009	[45]

* The percentage of relative level was calculated from 189 compounds detected with GC-MS and standard error (SE) was from three biological replications.

**Table 3:** Compositions of volatile blends collected in 6 h from aphid-fed chili pepper and cabbage plants that acted as attractants.

No.	Retention Time (min)	Compound	Molecular Formula	Chemical Formula	Relative Level ± SE (%) *	Reference
1	5.11	*α*-pinene (chili pepper)	C_10_H_16_	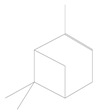	0.01 ± 0.009	[46]
2	9.99	Isophorone (cabbage)	C_9_H_14_O	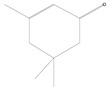	0.08 ± 0.006	[47]
3	12.19	Decanal (chili pepper)	C_10_H_20_O	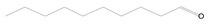	0.10 ± 0.009	[48]
4	39.34	Phthalic acid (chili pepper)	C_24_H_38_O_4_	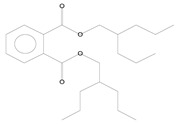	0.02 ± 0.006	[49]

* The percentage of relative level was calculated from 217 and 210 compounds detected with GC-MS for aphid-fed chili pepper and cabbage plants, respectively, and standard error (SE) was from three biological replications.

## Data Availability

The data are available from the author on reasonable request.

## Supplementary Materials

The following supporting information can be downloaded at: https://www.mdpi.com/article/10.3390/plants11101350/s1, Figure S1: Nylon mesh insect-rearing cage (40 cm × 40 cm × 30 cm) used to rear the pest (*Myzus persicae*) and its parasitoid (*Aphelinus varipes*), Figure S2: A schematic diagram representing the size of the filter paper used for the chemical attraction/repellent experiment; Table S1: Ten pairs of plants were tested in several combinations. Each combination was tested with sixty parasitoids (a total of 60 × 55 = 3300), and the single female was considered a replicate and employed only once in the entire experiment, Table S2: Four chemicals were tested in several combinations. Each combination was tested with sixty parasitoids (a total of 60 × 30 = 1800), and the single female was considered a replicate and employed only once in the entire experiment.
