# Supplementary material for: Plant Volatiles and Herbivore Induced Plant Volatiles from Chili Pepper Act as Attractant of the Aphid Parasitoid Aphelinus varipes (Hymenoptera: Aphelinidae)
Source: Plants (Basel). 2022 May 19;11(10):1350. doi: 10.3390/plants11101350 (PMC9145887; doi:10.3390/plants11101350)
Supplement: Supplementary file 1 [file plants-11-01350-s001.zip › plants-1692088-supplementary.pdf]

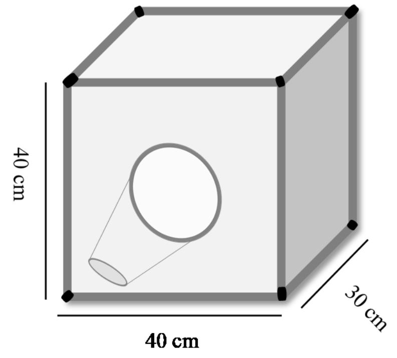

Figure S1. Nylon mesh insect rearing cage (40 cm × 40 cm × 30 cm) used to rear pest (*Myzus persicae*) and its parasitoid (*Aphelinus varipes*).

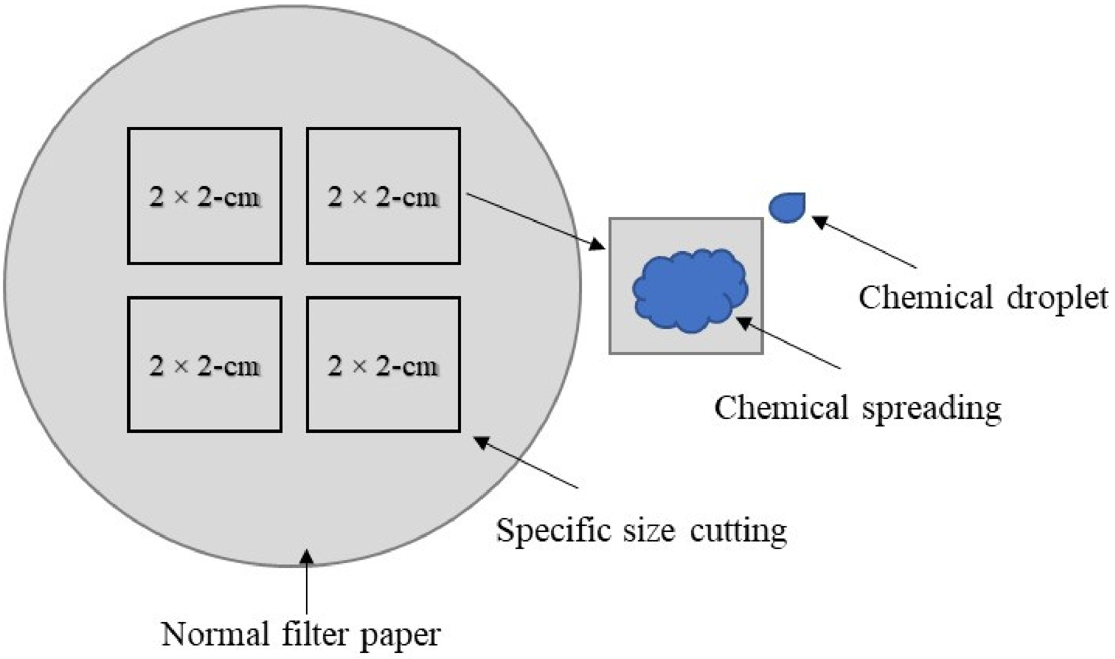

Figure S2. A schematic diagram representing the size of filter paper used for chemical attraction/repellent experiment.

Table S1. Ten pairs of plants were tested in several combinations. Each combination was tested with sixty parasitoids (A total of 60 × 55 = 3300), and single female was considered a replicate and was employed only once in the entire experiment.

Serial No.	Treatments		Tested Female Parasitoid
1	Clean air	Undamaged chili pepper	60
2	Clean air	Undamaged eggplant	60
3	Clean air	Undamaged crown daisy	60
4	Clean air	Undamaged Chinese cabbage	60
5	Clean air	Undamaged cabbage	60
6	Clean air	Chili pepper/aphid complex	60

7	Clean air	Eggplant/aphid complex	60
8	Clean air	Crown daisy/aphid complex	60
9	Clean air	Chinese cabbage/aphid complex	60
10	Clean air	Cabbage/aphid complex	60
11	Undamaged chili pepper	Undamaged egg plant	60
12	Undamaged chili pepper	Undamaged crown daisy	60
13	Undamaged chili pepper	Undamaged Chinese cabbage	60
14	Undamaged chili pepper	Undamaged cabbage	60
15	Undamaged egg plant	Undamaged crown daisy	60
16	Undamaged egg plant	Undamaged Chinese cabbage	60
17	Undamaged egg plant	Undamaged cabbage	60
18	Undamaged crown daisy	Undamaged Chinese cabbage	60
19	Undamaged crown daisy	Undamaged cabbage	60
20	Undamaged Chinese cabbage	Undamaged cabbage	60
21	Chili pepper/aphid complex	Undamaged chili pepper	60
22	Chili pepper/aphid complex	Undamaged eggplant	60
23	Chili pepper/aphid complex	Undamaged crown daisy	60
24	Chili pepper/aphid complex	Undamaged Chinese cabbage	60
25	Chili pepper/aphid complex	Undamaged cabbage	60
26	Eggplant/aphid complex	Undamaged chili pepper	60
27	Eggplant/aphid complex	Undamaged eggplant	60
28	Eggplant/aphid complex	Undamaged crown daisy	60
29	Eggplant/aphid complex	Undamaged Chinese cabbage	60
30	Eggplant/aphid complex	Undamaged cabbage	60
31	Crown daisy/aphid complex	Undamaged chili pepper	60
32	Crown daisy/aphid complex	Undamaged eggplant	60
33	Crown daisy/aphid complex	Undamaged crown daisy	60
34	Crown daisy/aphid complex	Undamaged Chinese cabbage	60
35	Crown daisy/aphid complex	Undamaged cabbage	60
36	Chinese cabbage/aphid complex	Undamaged chili pepper	60
37	Chinese cabbage/aphid complex	Undamaged eggplant	60
38	Chinese cabbage/aphid complex	Undamaged crown daisy	60
39	Chinese cabbage/aphid complex	Undamaged Chinese cabbage	60
40	Chinese cabbage/aphid complex	Undamaged cabbage	60
41	Cabbage/aphid complex	Undamaged chili pepper	60
42	Cabbage/aphid complex	Undamaged eggplant	60
43	Cabbage/aphid complex	Undamaged crown daisy	60
44	Cabbage/aphid complex	Undamaged Chinese cabbage	60
45	Cabbage/aphid complex	Undamaged cabbage	60
46	Chili pepper/aphid complex	Eggplant/aphid complex	60
47	Chili pepper/aphid complex	Crown daisy/aphid complex	60
48	Chili pepper/aphid complex	Chinese cabbage/aphid complex	60
49	Chili pepper/aphid complex	Cabbage/aphid complex	60
50	Eggplant/aphid complex	Crown daisy/aphid complex	60
51	Eggplant/aphid complex	Chinese cabbage/aphid complex	60
52	Eggplant/aphid complex	Cabbage/aphid complex	60

53	Crown daisy/aphid complex	Chinese cabbage/aphid complex	60
54	Crown daisy/aphid complex	Cabbage/aphid complex	60
55	Chinese cabbage/aphid complex	Cabbage/aphid complex	60
			**Total: 3300**

Table S2. Four chemicals were tested in several combinations. Each combination was tested with sixty parasitoids (A total of 60 × 30 = 1800), and single female was considered a replicate and was employed only once in the entire experiment.

Serial No.	Treatments		Tested Female Parasitoid
1	Clean air	α- pinene 1 ng/uL	60
2	Clean air	α- pinene 10 ng/uL	60
3	Clean air	α- pinene 100 ng/uL	60
4	Clean air	Phthalic acid 1 ng/uL	60
5	Clean air	Phthalic acid 10 ng/uL	60
6	Clean air	Phthalic acid 100 ng/uL	60
7	Clean air	Decanal 1 ng/uL	60
8	Clean air	Decanal 10 ng/uL	60
9	Clean air	Decanal 100 ng/uL	60
10	Clean air	Isophorone 1 ng/uL	60
11	Clean air	Isophorone 10 ng/uL	60
12	Clean air	Isophorone 100 ng/uL	60
13	α- pinene 1 ng/uL	α- pinene 10 ng/uL	60
14	α- pinene 1 ng/uL	α- pinene 100 ng/uL	60
15	α- pinene 100 ng/uL	α- pinene 100 ng/uL	60
16	Phthalic acid 1 ng/uL	Phthalic acid 10 ng/uL	60
17	Phthalic acid 1 ng/uL	Phthalic acid 100 ng/uL	60
18	Phthalic acid 10 ng/uL	Phthalic acid 100 ng/uL	60
19	Decanal 1 ng/uL	Decanal 10 ng/uL	60
20	Decanal 1 ng/uL	Decanal 100 ng/uL	60
21	Decanal 10 ng/uL	Decanal 100 ng/uL	60
22	Isophorone 1 ng/uL	Isophorone 10 ng/uL	60
23	Isophorone 1 ng/uL	Isophorone 100 ng/uL	60
24	Isophorone 10 ng/uL	Isophorone 100 ng/uL	60
25	Phthalic acid 100 ng/uL	α- pinene 100 ng/uL	60
26	Decanal 100 ng/uL	α- pinene 100 ng/uL	60
27	Decanal 100 ng/uL	Phthalic acid 100 ng/uL	60
28	Isophorone 100 ng/uL	α- pinene 100 ng/uL	60
29	Phthalic acid 100 ng/uL	Isophorone 100 ng/uL	60
30	Decanal 100 ng/uL	Isophorone 100 ng/uL	60
			**Total: 1800**
